# A study on the exploration of mild cognitive impairment in Parkinson’s disease based on decision-making cognitive computing

**DOI:** 10.3389/fnins.2024.1495975

**Published:** 2025-01-07

**Authors:** Shouqiang Huang, Kai Li, Chen Wang, Jiakang Liu, Shuwu Li, Yuting Tu, Bo Wang, Huangqin Feng, Qin Yu, Hongzhou Lin, Yuzhe Xu, Jinghang Wu, Ting Zhang, Tong Chen

**Affiliations:** ^1^School of Medical Technology and Information Engineering, Zhejiang Chinese Medical University, Hangzhou, China; ^2^School of Information Engineering, Hangzhou Medical College, Hangzhou, China; ^3^Zhejiang Engineering Research Center for Brain Cognition and Brain Diseases Digital Medical Instruments, Hangzhou Medical College, Hangzhou, China; ^4^Department of Neurology, The Second Medical Center and National Clinical Research Center for Geriatric Diseases, Chinese PLA General Hospital, Beijing, China

**Keywords:** exploration, Parkinson’s disease, mild cognitive impairment, decision-making, digital biomarkers

## Abstract

Mild cognitive impairment in Parkinson’s disease (PD-MCI) as an independent risk factor for dementia in Parkinson’s disease has prognostic value in predicting dementia in PD patients. It was found that the calculation of cognitive function decision-making could better evaluate the cognitive function of PD-MCI. Therefore, this study explored deficits in decision-making cognitive function in PD-MCI population, and mined novel digital biomarkers for recognizing early cognitive decline in PD-MCI through an independently designed maze decision-making digital assessment paradigm. This study included 30 healthy controls 37 PD with normal cognition (PD-NC) and 40 PD-MCI patients. Through difference comparison and stepwise regression analysis, two digital decision-making biomarkers, total decision time and performance average acceleration, were screened, and their joint area under curve for the ability to discriminate between PD-MCI and PD-NC was 0.909, and for the ability to discriminate between PD-MCI and NC was 0.942. In addition, it was found that maze digital decision-making biomarkers had greater early warning efficacy in men than in women. Unlike traditional methods, this study used digital dynamic assessment to reveal possible decision-making cognitive deficits in the PD-MCI populations, which provides new ideas for effective screening for PD-MCI.

## 1 Introduction

Parkinson’s disease (PD) is the second most common neurodegenerative disease after Alzheimer’s disease. It causes a progressive clinical course characterized by premotor, nonmotor, and motor symptoms by affecting dopaminergic neurons in the substantia nigra of the midbrain (Weintraub et al., 2022). Non-motor symptoms can occur in the premotor phase of PD. The non-motor symptoms of PD have received increasing attention from scholars, and among these non-motor symptoms, cognitive dysfunction, especially mild cognitive impairment (MCI), has gradually become a hot topic of current research. PD-MCI is a transitional stage between PD with normal cognitive function and PD dementia. One study found that 19–62% of patients with PD-MCI progressed to PD dementia (PDD) by the time they were followed up 2–5 years after diagnosis (Wood et al., 2016). Another 5-year cohort follow-up study from the Norwegian Movement Disorder Center demonstrated that the conversion rate to PD dementia in patients with PD-MCI picked up to be 39–50% (Pedersen et al., 2017). Additionally, one study demonstrated that the odds of patients with PD-MCI progressing to dementia each year were 6–15% (Monastero et al., 2018). PD-MCI, as an early stage of PD dementia, is an independent risk factor for progression to PD dementia (Baiano et al., 2020). PD-MCI, whether it persists or reverts to normal cognition, has prognostic value in predicting dementia in PD (Pedersen et al., 2017). Therefore, exploratory studies focusing on PD-MCI populations are clinically important in studying the development of PD disease.

Currently, methods for early identification of PD-MCI based on cognitive function assessment include clinical neuropsychological testing and biomarker detection. Given the diversity of cognitive impairment patterns in patients with PD-MCI, which primarily involves impaired cognitive function in executive, visuospatial, attention, and memory. Therefore, neuropsychological testing requires multiple single-domain or global cognitive function screening scales for assessment, but this process is subjective, time-consuming, and of low sensitivity. In recent years, neuroimaging techniques such as magnetic resonance imaging and positron emission computed tomography have evolved into powerful complementary tools for diagnosing PD-MCI (Devignes et al., 2022); However, neuroimaging techniques are not only costly and poorly reproducible, but also need to be performed in specialized medical institutions. Therefore, diagnostic methods based on neuroimaging analysis are still unable to meet the demand for broad early identification of PD-MCI. Given the current limited methods for early identification of PD-MCI and the global shortage of neurologists (Burton, 2018), there is an urgent need to find new ideas for early identification of PD-MCI.

Decision-making serves as a window into cognition (Shadlen and Kiani, 2013). Recently, several studies have also confirmed that decision-making tasks can well characterize cognitive deficits and revealed that decision-making deficits are the result of abnormalities in connectivity across multiple brain regions (Stern, 2022; Thiebaut de Schotten and Forkel, 2022; Jian et al., 2024; Kuan et al., 2024). Decision-making is a high-level cognitive process of evaluating and choosing between alternatives that involves deliberate deliberation, planning, and strategizing, which is well characterized as computationally and interpretatively tractable. Research has found that decision-making processes often involve the participation of multiple cognitive functions (Schiebener et al., 2014; Bruine de Bruin et al., 2007). A review concluded that decision-making is a complex mental function that is influenced by multiple cognitive and behavioral processes such as visuospatial, executive, and memory (Gleichgerrcht et al., 2010). For example, in a maze path decision task, where subjects are required to make a path prediction through visual search or selective attention, executive abilities, visuospatial memory abilities are involved (Thomas et al., 2014; Parrini et al., 2024; Lee and Kaang, 2010). In ambiguous and risky decision-making tasks, stronger executive inhibitory control and working memory are often involved in processing information and inhibiting irrelevant responses (Colautti et al., 2022). Thus, decision-making serves as a window into cognition and may better characterize cognitive decline.

Currently, several studies provide early detection of MCI based on decision-making tasks. One study based on a virtual spatial navigation task reflected the presence of deficits in decision-making ability for path planning in early risk carriers of AD (Bierbrauer et al., 2020); a meta-analysis showed that spatial navigation decision-making ability was well assessed in the MCI population using a maze test (Plácido et al., 2022); and results of a recent meta-analysis showed that patients with MCI have mild or moderate impairments in decision-making abilities related to financial management, medical adherence, specific cognitive performance, hazardous conditions, and especially uncertain living situations (Alfeo et al., 2024). Cognitive impairment in PD patients is mainly related to executive function, visuospatial function, attention, and memory. In recent years, studies have also found that declines in decision-making abilities are also often observed in people with PD. One study found that PD populations have difficulty utilizing previously learned information to guide their decisions during perceptual decision-making (Perugini et al., 2016), and another study revealed that PD populations exhibit impaired integration of memory and sensory information during perceptual decision-making (Perugini et al., 2018). In addition, in the early cognitive impairment stage of PD, studies have found that decision-making ability is already impaired in PD-MCI patients and is further impaired as these patients develop dementia (Martin et al., 2008). Another study characterizing early cognitive changes in Parkinson’s disease highlights the fact that Parkinson’s disease produces very specific and early cognitive changes in complex cognitive control, decision-making, and learning processes (Claassen and Wylie, 2012). For example, one study recruited a large number of early, nondemented PD patients for the Iowa Gambling Task Decision Making Study, which found that decision-making deficits were present in early, nondemented PD patients compared to normal control (NC) subjects (Kobayakawa et al., 2008). Thus, studying decision-making tasks in PD-MCI populations may provide a better exploration of cognitive deficits in PD-MCI populations, and may also hopefully lead to the early identification of PD-MCI populations.

Digital biomarkers have shown good potential in the field of PD-MCI population screening research. It characterizes and quantifies the early characteristics of the PD-MCI population through a variety of smart devices for rapid screening identification of the PD-MCI population (Brien et al., 2023; Lolekha et al., 2021; Liu et al., 2021). The maze test is a versatile cognitive functioning assessment tool that comprehensively evaluates an individual’s cognitive functioning, such as executive, visuospatial, and memory abilities, involved in maze-path decision-making tasks. Pre-existing maze tasks have been extensively tested in the field of PD screening, and some studies have also applied digital maze tasks to patients with cognitive impairment in PD. For example, one study recorded relevant cognitive digital biomarkers in PD patients through the Kiel motor maze to explore human spatial behavioral characteristics in the maze (Zeng et al., 2003). Another study was based on a self-developed maze-based puzzle game to assess cognitive and motor functions in neurodegenerative disorders, such as PD (Nef et al., 2020). In addition, a digital virtual water maze test was designed in a study to identify PD-MCI through extracted digital biomarkers related to heading error, path length, and latency to locate the target (Schneider et al., 2017). Thus, digital assessment based on maze tasks may be a new way to quickly and intelligently identify of cognitive deficits in decision-making in PD-MCI.

In summary, this study proposed to explore the decision-making cognitive deficits in the PD-MCI population and independently designed a novel method of decision-making dynamic digital assessment, which can capture the decision-making information of the natural and dynamic characteristics of the visual environment of PD-MCI patients and characterize the decision-making cognitive deficits in the PD-MCI population at a fine-grained level by using the maze decision-making digital assessment paradigm. Through preliminary clinical validation, the AUC of the screening efficacy of the method was 0.909 for PD-MCI and PD-NC, and 0.942 for PD-MCI and NC, further supporting that cognitive function deficits in decision-making are expected to be quantified as a novel digital biomarker of early cognitive decline in PD-MCI.

## 2 Materials and methods

### 2.1 Paradigms of human-computer interaction and the design of digital biomarkers

#### 2.1.1 Experimental paradigm design hardware and software conditions

**FIGURE 1:**
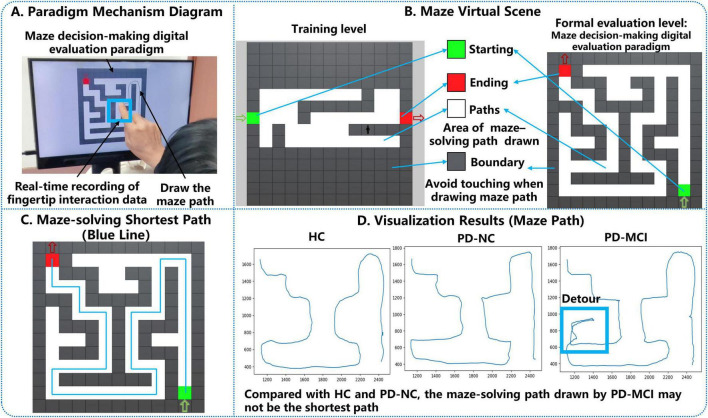
Paradigm introduction.

The hardware required for this experiment consists of an Intel computer (NUC11PAHi5), a touchable monitor (length, width and height of 392 × 250 × 10 mm, screen size of 17.3 inches, resolution of 3840 × 2160 pixels). The software system involved in this experiment is a human-computer interaction system. The front-end interface of the system is built through Electron and Vue3, the maze decision-making digital evaluation paradigm is built through Unity3D and integrated into the HCI system, the sampling frequency of the fingertip interaction data is about 50 Hz, the back-end system is built through python, and the fingertip interaction database is built through Mysql database. The paradigm mechanism diagram is shown in Figure 1A.

#### 2.1.2 Experimental paradigm design and principle interpretation

In the maze path decision-making task, subjects need to make path judgments through visual search or selective attention, which involves a variety of cognitive abilities such as planning, execution, and visuo-spatial (Thomas et al., 2014; Parrini et al., 2024; Lee and Kaang, 2010). Although the maze task has been extensively tested on PD patients, digitization of the maze task and the quantification of the maze task process at a fine-grained level throughout the entire process and its use in exploring cognitive dysfunction, there is a gap in relevant research. We collected human-computer interaction data that could reflect the planning process and performance process of the subjects through a human-computer interaction system, and thus assessed the cognitive functioning of the subjects in decision-making during the paradigm assessment.

The design of the Parkinson’s disease maze decision-making digital evaluation paradigm is as follows: In this study, we refer to the maze generation website^1^ created by John Lauro and other researchers at the University of Michigan as the maze layout generator, and we also refer to the relevant studies that have used the maze test to assess the cognitive status of middle-aged and elderly people with neurodegenerative diseases, and select the corresponding parameters to be input into the maze generator (Nef et al., 2020). Based on previous studies and daily life observations (Li et al., 2022), the majority of the subjects were right-handed, so the maze was chosen to run from the lower right entrance to the upper left exit so that the subject’s vision would not be affected during the operation. As a common tool for testing cognitive ability, spatial memory, and learning ability, mazes have appropriate design criteria (McClendon, 2001). In this study, based on the design principles of mazes (Bellot et al., 2021), the maze scenario of the Parkinson’s disease maze decision-making digital evaluation paradigm is shown in Figure 1B.

The goal and rules of the paradigm are that subjects need to use their right index finger to draw the shortest maze path from the starting point of the green square in the lower-right corner to the end point of the red square in the upper-left corner of the white grid (the area that can be touched by the finger when drawing maze paths) as soon as possible in the shortest possible time on the screen of the display screen, the shortest maze path can be seen in Figure 1C, and try not to touch the gray wall (the area that can be avoided when drawing maze paths with the finger). If you touch the gray wall, a red rectangle will appear to indicate the exact location of the gray wall. If the subject draws the path to solve the puzzle within 210 s, i.e., from the starting point of the green square in the lower right corner to the end point of the red square in the upper left corner, the experiment is regarded as a success. If the subject fails to draw the puzzle path within 210 s, the paradigm will end automatically and the experiment will be considered a failure. At the same time, in order to facilitate subjects to familiarize themselves with the paradigm objectives, we also set up a training level, which was designed after the Parkinson’s disease maze decision-making digital evaluation paradigm, including the color of the overall scene, the starting point and the end point of the markers, etc., but the difficulty of the training level will be lower than that of the formal evaluation level, which is reflected in the shortest paths in the training level are easier to find than that of the formal evaluation level and there are no more than one The shortest paths in the training level were easier to find than in the formal evaluation level, and there were no multiple forks in the road to interfere with subjects, as shown in Figure 1D.

#### 2.1.3 Definition and quantitative analysis of digital decision-making biomarkers

We extracted digital decision-making biomarkers via python (version 3.10.14) based on the above objectivized assessment data. In order to quantify subjects’ decision-making ability at a fine-grained level, we categorized the digital decision-making biomarkers into two dimensions: decision-making global digital biomarkers and decision-making process digital biomarkers, where the digital biomarkers for process decision-making were categorized into decision-making planning process digital biomarkers and decision-making performance process digital biomarkers.

Digital decision-making biomarkers are holistic evaluations of subjects in the maze decision-making digital evaluation paradigm, including subjects’ Total Decision Time and Decision Process Time, both of which characterize subjects’ overall performance in completing the paradigm.

Decision-making process digital biomarkers are process evaluations of specific decision-making behaviors of subjects during the maze decision-making digital assessment paradigm, which can be subdivided into decision-making planning process digital biomarkers and decision-making performance process digital biomarkers.

The decision-making planning process digital biomarkers are used to record the subjects’ planning and thinking about the maze solution paths before or during the execution process through the fingertip interaction technology, including the subjects’ Initial planning time and Performance pause time, which is used to characterize the subjects’ observation of the overall scene of the maze and the planning and thinking of choosing the shortest solution paths during the paradigm evaluation process. The planning and thinking of the shortest path to solve the maze was characterized.

The decision-making performance process digital biomarkers are recorded by the fingertip interaction technology to record the subjects’ execution after planning, including Performance path length, Performance error path length, Number of performance errors, Performance average speed, Performance speed variability, Initial performance acceleration, and Performance average acceleration. The Performance path length is used to characterize the length of the puzzle path drawn by the subject, the Performance error path length is used to characterize the length of the path drawn by the subject for the wrong puzzle, the Number of performance errors is used to characterize the situation in which the subject touches the wall, the Performance average speed is used to characterize the situation in which the drawing speed is used during the process of the subject’s drawing the puzzle path, the execution speed variability is used to characterize the situation in which the drawing speed is used in the process of the subject’s drawing the puzzle path. The Initial performance acceleration and Performance average acceleration are used to characterize the variation of the drawing speed during the drawing of the puzzle path.

To facilitate the subsequent digital biomarker mining analysis, we provide a detailed conceptual definition of the various digital biomarkers in the paradigm:

(1) The group of digital biomarkers for global decision-making

**FIGURE 2:**
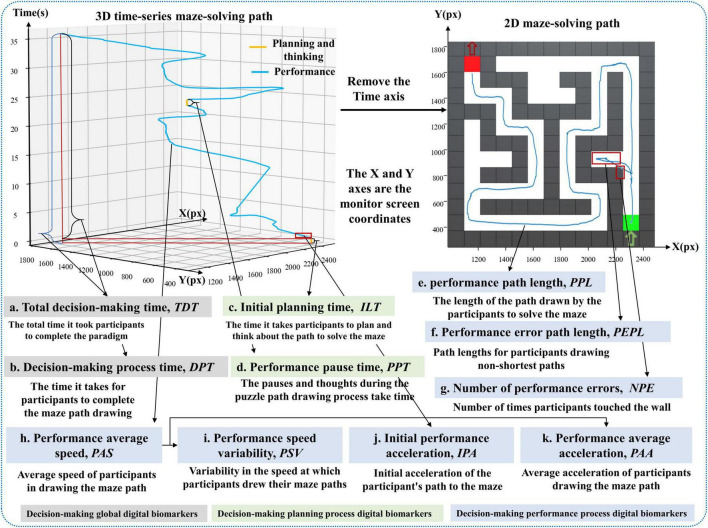
Illustration of digital biomarker for global decision-making, decision-making planning process digital biomarkers and decision-making performance process digital biomarkers.

An overview of the digital biomarker for global decision-making is shown in Table 1. A graphical representation of the digital biomarker for global decision-making is shown in Figure 2.

**TABLE 1:** Decision-making global digital biomarkers.

Serial number	Digital biomarker	Abbreviation	Unit
a	Total decision-making time	*TDT*	second, s
b	Decision-making process time	*DPT*	second, s

a. Total decision time (*TDT*): Calculate the time interval from the time the subject entered the paradigm to the time he/she finished drawing the puzzle path with his/her finger, i.e., the total time taken to complete the paradigm (seconds, s).

b. Decision process time (*DPT*): Calculate the time interval from the start of the green square in the lower right corner to the end of the red square in the upper left corner to complete the maze path drawing with the finger of the subject in the paradigm evaluation process (seconds, s).

(2) The group of digital biomarkers for process decision-making

① Decision-making planning process digital biomarkers

An overview of the decision-making planning process digital biomarkers is shown in Table 2. A graphical representation of the decision-making planning process digital biomarkers is shown in Figure 2.

**TABLE 2:** Decision-making planning process digital biomarkers.

Serial number	Digital biomarker	Abbreviation	Unit
c	Initial planning time	*ILT*	second, s
d	Performance pause time	*PPT*	second, s

c. Initial planning time (*ILT*): calculates the time interval between when the subject enters the paradigm and when he/she first touches the starting point of the green square in the lower right corner with his/her finger (seconds, s).

d. Performance pause time (*PPT*): calculates the cumulative time that the subject’s finger stays still while drawing the puzzle path during the paradigm evaluation (seconds, s).

② Decision-making performance process digital biomarkers

An overview of the decision-making performance process digital biomarkers is shown in Table 3. A graphical representation of the decision-making performance process digital biomarkers is shown in Figure 2.

**TABLE 3:** Decision-making performance process digital biomarkers.

Serial number	Digital biomarker	Abbreviation	Unit
e	Performance path length	*PPL*	pixel, px
f	Performance error path length	*PEPL*	pixel, px
g	Number of performance errors	*NPE*	times
h	Performance average speed	*PAS*	px/s
i	Performance speed variability	*PSV*	/
j	Initial performance acceleration	*IPA*	px/s^2^
k	Performance average acceleration	*PAA*	px/s^2^

e. Performance path length (*PPL*): calculates the total length of the path drawn by the subject to solve the maze during the paradigm evaluation (pixels, px).

f. Performance error path length (*PEPL*): calculates the length of the path drawn by the subject’s finger on the white grid of the non-shortest path during the paradigm evaluation (pixels, px).

g. Number of performance errors (*NPE*): calculates the number of times that the subject’s finger touched the gray wall while drawing the puzzle path during the paradigm evaluation (times).

h. Performance average speed (*PAS*): calculates the average drawing speed of the subject’s finger during the paradigm evaluation (pixels/second, px/s).

i. Performance speed variability (*PSV*): calculates the speed variation of the finger drawing the puzzle path, i.e., the speed coefficient of variation, of the subject during the paradigm evaluation (pixels/second, px/s).

j. Initial performance acceleration (*IPA*): calculates the acceleration value at the first three drawn path sampling points of the subject during the paradigm evaluation (pixels/second, px/s).

k. Performance average acceleration (*PAA*): calculates the average acceleration value of the finger-drawn puzzle paths of the subjects during the paradigm evaluation (pixels/second squared, px/s^2^).

The relevant extraction algorithms for the above digital decision-making biomarkers are as follows:

We know the subjects’ Total decision-making time (*TDT*) (TDT < 210 s) and Initial planning time (*ILT*), and the Decision-making process time (*DPT*) is calculated as:


(1) D⁢P⁢T=T⁢D⁢T-I⁢L⁢T

The execution trajectory coordinate point sampling frequency (SF) is 44 Hz (i.e., 0.022 s is collected once), the number of execution trajectory coordinate samples during the paradigm evaluation process is *I* (*I = DPT × SF*), the execution trajectory coordinate point corresponding to the *i^th^* sampling point is (X_*i*_, Y_*i*_) (0 < *i* ≤ *I*, *i*∈N), and the time interval between any two neighboring execution trajectory coordinate points is *t*_*i*_, which is calculated as follows:


(2) ti=1S⁢F(0<i≤I-1,i∈N)*

We compute the Performance pause time (*PPT*) by determining whether the neighboring execution trajectory coordinate points are the same, which is computed as:


(3) $$j\,u\,d\,g\,e\,\left(i\right)=\left\{ \begin{array}{c} 1\left(X_{i+1}=X_{i}andY_{i+1}=Y_{i}\right)\\ 0\left(X_{i+1}\neq X_{i}orY_{i+1}\neq Y_{i}\right) \end{array}\right.$$

(4) PPT=∑i=1i=I-1j⁢u⁢d⁢g⁢e⁢(i)S⁢F(0<i≤I-1,i∈N*)

In Equation (3), *judge*(*i*)is used to determine whether the neighboring decision-making performance trajectory coordinate points are the same.

The distance between two neighboring sampling execution trajectory coordinate points is *D_*i*_*,


(5) Di=(Xi+1-Xi)2+(Yi+1-Yi)2(0<i≤I-1,i∈N*)

In Equation (5), *X*_*i*+1_ is the horizontal coordinate of the (*i*+1)*^th^* execution trajectory coordinate point, and *Y*_*i*+1_ is the vertical coordinate of the (*i*+1)*^th^* execution trajectory coordinate point.

The Performance path length (*PPL*) is the cumulative sum of the distances *D*_*i*_ of the neighboring execution trajectory coordinate points for each time of the maze, which is calculated by the formula:


(6) PPL=∑i=1i=I-1Di(0<i≤I-1,i∈N*)

Since the shortest path of the maze (the optimal decision-making performance path out of the maze) has uniqueness, the shortest path region of the maze is set as φ. The execution trajectory coordinate points collected in the region outside the region φ are the error trajectory coordinate points (*EX_*i*_, EY_*i*_*), and the distance between two neighboring error trajectory samples is set as *ED*_*i*_, which is computed by the formula:


(7) $$E\,D_{i}=\sqrt{{\left(E\,X_{i+1}-E\,X_{i}\right)}^{2}+{\left(E\,Y_{i+1}-E\,Y_{i}\right)}^{2}}$$

(1≤i≤I-1,i∈N*)

The Performance error path length (*PEPL*) is computed as


(8) PEPL=∑i=1i=I-1EDi(1≤i≤I-1,i∈N*)

The Performance average speed (*PAS*) is calculated as:


(9) $$P\,A\,S=\frac{P\,P\,L}{T\,D\,T}$$

We calculate the velocity *V*_*i*_ between every two neighboring execution trajectory coordinate points, and thus the Performance speed variability (*PSV*), the Initial performance acceleration (*IPA*), and the Performance average acceleration (*PAA*), respectively, with the formula:


(10) Vi=Diti(0<i≤I-1,i∈N*)

(11) $$\sigma_{V}=\sqrt{\frac{\sum_{\text{i}=1}^{I-1}V_{i}-P\,A\,S^{2}}{\text{n}}}$$

(12) $$P\,S\,V=\sigma_{V}/P\,A\,S$$

(13) $$I\,P\,A=\frac{\left|V_{1}-V_{2}\right|}{t_{1}+t_{2}}$$

(14) $$P\,A\,A=\sum_{i=1}^{i=I-1}\frac{\left|V_{i}-V_{i+1}\right|}{t_{i}+t_{i+1}}$$

In Equation (11), σ_*V*_ is the standard deviation of the execution speed.

#### 2.1.4 Experimental rule design

Prior to the official start of the Parkinson’s Disease maze decision-making digital evaluation paradigm, subjects will undergo a training level to familiarize themselves with the paradigm process and manipulation methods. During the training level, each subject can repeat the process as many times as he/she wants and there is no time limit. In the Parkinson’s disease maze decision-making digital evaluation paradigm, subjects were required to use their right index finger to complete the maze path drawing, but each subject could only do it once, and the maze scenes were all the same, so before the Parkinson’s disease maze decision-making digital evaluation paradigm formally began, subjects were allowed to perform the training level several times, and it was repeated to confirm that the subjects were able to complete the experiments, and if the subject’s experiments were unsuccessful, the data related to the subject would not be analyzed in the subsequent analyses. Subjects’ relevant data will be analyzed. Meanwhile, all subjects included in this experiment were right-handed, and the affected sides of PD-MCI patients were all on the right side, to avoid interference of experimental results with hand habits.

### 2.2 Experimental setup

#### 2.2.1 Recruitment of subjects

117 subjects were recruited in this study, of which 8 were not included in the trial because they did not meet the inclusion criteria. 109 subjects were included in the trial, including 41 PD-MCI patients, 37 PD-NC patients and 31 age-matched healthy controls (HC), who were included in the PD-MCI group, PD-NC group and the HC group, respectively. All subjects were recruited through the Department of Neurology and the Department of Nuclear Medicine of the First Medical Center of the PLA General Hospital and diagnosed by neurologists. All experimental procedures were in accordance with the Declaration of Helsinki and approved by the Medical Ethics Committee of the PLA General Hospital (Ethics Number: S2022-770-02). Subjects were given written informed consent. The specific inclusion criteria are as follows:

Inclusion criteria for PD-MCI: (1) Primary PD was diagnosed according to the brain bank of the PD Association in London, UK (Hughes et al., 1992); (2) Slow cognitive decline reported by the patient information providers or observed by the clinicians; (3) Cognitive decline reflected in formal neuropsychological tests or overall cognitive performance scales; (4) Cognitive decline will not result in dependence on the abilities to live and work, although there may be minor impairments in performing difficult tasks; (5) Patient was able to cooperate in completing the maze decision-making digital evaluation paradigm, right-handed; (6) Patient was aged 45–80 years, regardless of gender; (7) Signing an informed consent form.

Exclusion Criteria for PD-MCI: (1) PD dementia diagnosed according to the guidelines proposed by the working group of the MDS; (2) Cognitive dysfunction caused by delirium, cerebral infarction, cerebral hemorrhage, subarachnoid hemorrhage, severe depression, metabolic disorders, medication side effects, or head trauma; (3) Other PD-related co-morbidities considered by the clinician to have a significant impact on cognitive testing (e.g., movement disorders or severe anxiety, depression, daytime excessive somnolence, or psychosis); (4) PD-MCI with cognitive impairments that prevent them from completing paradigmatic tasks.

Inclusion criteria for PD-NC: (1) Primary PD was diagnosed according to the brain bank of the PD Association in London, UK (Hughes et al., 1992); (2) Cognitive decline not reported by the patient information provider or observed by the clinician; (3) Cognitive decline not reflected in formal neuropsychological tests or overall cognitive scales; (4) Patient was aged 45–80 years, regardless of gender; (5) Signing an informed consent form.

Exclusion criteria for PD-NC: (1) Those who underwent surgical implantation of electrodes to give deep brain stimulation therapy; (2) Concomitant diseases with other central nervous system damage (e.g., metabolic encephalopathy, immune encephalopathy, cranio-cerebral trauma, etc.); (3) exclude superimposed Parkinson’s disease syndrome (e.g., multi-systemic atrophy, progressive supranuclear palsy, corticobasal ganglionic degeneration, etc.) and secondary Parkinson’s syndrome (if there is ischemic cerebrovascular disease, encephalitis, poisoning, tumor, trauma, heredity, etc.); (4) Exclude primary or organic mental illnesses such as schizophrenia, depression, excessive daytime somnolence, sleep attacks, etc.; (5) Those with alcohol dependence or drug addiction.

Inclusion criteria for healthy controls: (1) No complaints and objective evidence of neurological disease (normal neurological clinical examination); (2) No history of REM sleep disorder; (3) Age, gender, and literacy level consistent with PD-MCI; (4) Patient’s ability to cooperate in the examination and paradigm task, right-handed; (5) Signing an informed consent form.

During the formal experiment, one subject in the PD group refused to participate due to exacerbation and one subject in the HC group withdrew for other reasons. The final valid sample size was 107 individuals, including 40 PD-MCI in the PD-MCI group, 37 PD-NC in PD-NC group, and 30 age-matched healthy controls in the HC group. To ensure the consistency of all data, in this study, the PD-MCI and PD-NC underwent the above paradigm evaluation without taking levodopa-based medications in the morning of the same day. Meanwhile, the MDS Unified-Parkinson Disease Rating Scale (MDS-UPDRS) scores, MMSE scores and MoCA scores were administered to 107 subjects by neurological assessors before the paradigm evaluation.

#### 2.2.2 Experimental procedure

All subjects conducted the experiment in a quiet room to exclude noisy environmental factors from interfering with subjects’ task performance. We placed a comfortable and stable chair 90 cm in front of the interactive display. The distance between the subject and the interactive display was approximately 40 cm, ensuring that subjects could both see the screen and easily draw the puzzle path on the screen with their fingers. Visual distractions or manipulation inconveniences were ruled out as interfering with subjects’ task performance. Subjects were first given a training session, and once they were fully familiar with the instructions and objectives, they were given the Parkinson’s Disease maze decision-making digital evaluation paradigm, in which subjects were required to use the index finger of their right hand to draw the shortest maze path as quickly as possible on a white grid (the area that can be touched by the finger when drawing the maze path), from the start of the green square in the lower-right corner to the end of the red square in the upper-left corner, without touching the gray square, and without touching the gray square. Try not to touch the gray wall (the area that is not touchable when drawing the maze path with your finger), the paradigm time limit is 210 s.

### 2.3 Statistical analysis

All statistical analyses were performed using the SPSS 26.0 software package for general data analysis. Measures conforming to normal distribution were expressed as x ± s, and measures conforming to skewed distribution were expressed as M(IQR). When multiple sample group comparisons were involved, if the sample data all conformed to normal distribution and the variance was homogeneous, one-way ANOVA was used, and when there was a difference, the comparison groups were compared one by one by using the LSD method; if the sample data of the multiple groups did not all conform to normal distribution, the Kruskal-Wallis test was used and when there was a difference, the Bonferroni method was used to compare the comparison groups one by one. Qualitative information was expressed as a rate (%) and differences between groups were compared by the chi-square test. Next, binary logistic stepwise regression was applied to screen digital decision-making biomarkers. Finally, we used a binary logistic regression model to draw the receiver operating characteristic (ROC) curve and adopted the area under the curve (AUC) to evaluate the effectiveness of a single decision-making digital biomarker and a combination of multiple digital decision-making biomarkers in PD-MCI warning. *P* < 0.05 were considered statistically significant.

## 3 Results

### 3.1 Demographic and clinical characteristics

A total of 107 subjects aged 45–85 years, including 30 normal elderly, 37 PD-NC patients, and 40 PD-MCI patients, were enrolled in this study, and they were included in the HC group, the PD-NC group, and the PD-MCI group, respectively. We analyzed the difference in baseline information of the three groups, and the results of the difference analysis of the three groups of subjects in terms of age, gender, years of education, MMSE scale, MoCA scale, and MDS-UPDRS-III scale are shown in Table 4.

**TABLE 4:** Distribution of demographic and clinical characteristics of the HC, PD-NC and PD-MCI groups.

	HC *n* = 30	PD-NC *n* = 37	PD-MCI *n* = 40	HC *VS.* PD-NC_1_	HC *VS.* PD-MCI_1_	PD-NC *VS.* PD-MCI	HC *VS.* PD-NC *VS.* PD-MCI
				***p*-value**			
Age, years	66.40 ± 10.25	63.70 ± 6.99	66.50 (10.25)	>0.05	>0.05	>0.05	0.268
Sex (female/male)	20/10	16/21	18/22	>0.05	>0.05	>0.05	0.118
Years of education	9.23 ± 3.43	12.00 (6.00)	11.50 (5.50)	>0.05	>0.05	>0.05	0.120
MMSE score	26.50 (4.00)	28.00 (1.50)	25.00 (2.00)	0.084	<0.001**	<0.001**	<0.001**
MoCA score	24.50 (2.25)	25.00 (1.50)	22.00 (3.00)	0.317	<0.001**	<0.001**	<0.001**
UPDRS-III score	4.00 (2.25)	15.00 (4.00)	18.50 (11.00)	<0.001**	<0.001**	0.130	<0.001**

**indicates a significant difference between the two groups, at *p* < 0.01.

### 3.2 Analysis of digital biomarkers

We compared the digital decision-making biomarkers in the HC group, the PD-NC group, and the PD-MCI group. We found that a total of 8 digital decision-making biomarkers were significantly different between groups (p < 0.05). In PD-MCI and PD-NC groups, Total decision-making time (*TDT*), Decision-making process time (*DPT*), Performance path length (*PPL*), Performance pause time (*PPT*), Performance error path length (*PEPL*) in PD-MCI was larger than that in PD-NC group, and Performance average speed (*PAS*), Performance average acceleration (*PAA*) in PD-MCI was smaller than that in PD-NC group; In PD-MCI and HC groups, Total decision time (*TDT*), Decision-making process time (*DPT*) was greater in the PD-MCI group than in the HC group, and Performance average speed (*PAS*), Performance average acceleration (*PAA*), Performance speed variability (*PSV*)was less in the PD-MCI than in the HC group; In PD-NC and HC groups, Performance pause time (*PPT*), Performance error path length (*PEPL*) was smaller in the PD-NC group than in the HC group. The results of the specific digital decision-making biomarkers variability analysis were shown in Table 5.

**TABLE 5:** Intergroup variability of digital decision-making biomarkers of the HC, PD-NC and PD-MCI groups.

	HC *n* = 30	PD-NC *n* = 37	PD-MCI *n* = 40	HC *VS.* PD-NC	HC *VS.* PD-MCI	PD-NC *VS.* PD-MCI	HC *VS.* PD-NC *VS.* PD-MCI
				***p*-value**			
**Decision-making global digital biomarkers**							
*TDT, s*	31.16 ± 13.47	24.40 (9.99)	42.54 (20.51)	0.570	<0.001**	<0.001**	<0.001**
*DPT, s*	26.71 (20.59)	22.85 (20.59)	41.45 (18.79)	1.000	<0.001**	<0.001**	<0.001**
**Decision-making planning process digital biomarkers**							
*ILT, s*	0.03 (0.02)	0.02 (0.02)	0.02 (0.02)	>0.05	>0.05	>0.05	0.573
*PPT, s*	0.97 (3.04)	0.24 (0.72)	2.29 (2.16)	0.008**	0.053	<0.001**	<0.001**
**Decision-making performance process digital biomarkers**							
*PPL, px*	6788.59 (1887.11)	6638.70 (608.15)	6927.17 (1148.83)	0.106	1.000	0.008**	0.008**
*PEPL, px*	0.00 (1074.82)	0.00 (0.00)	0.00 (514.79)	0.026*	1.000	0.033*	0.011*
*NPE, times*	2.50 (6.00)	1.00 (6.00)	1.00 (5.00)	>0.05	>0.05	>0.05	0.264
*PAS, px/s*	279.48 (176.79)	286.38 (137.11)	172.08 (76.59)	1.000	<0.001**	<0.001**	<0.001**
*PSV*	368.65 (330.63)	304.18 (158.69)	235.90 (143.23)	0.080	0.001**	0.503	0.002**
*IPA, px/s2*	0 (1896.86)	778.19 (4540.36)	308.43 (1877.84)	>0.05	>0.05	>0.05	0.105
*PAA, px/s2*	8222.62 (4537.96)	7943.96 (4969.35)	4144.64 (1416.88)	1.000	<0.001**	<0.001**	<0.001**

*Indicates a significant difference between the two groups, at 0.01 < *p* < 0.05;

**indicates a significant difference between the two groups, at *p* < 0.01.

### 3.3 ROC curves for identifying PD-MCI from all subjects

We plotted the ROC curves for HC and PD-NC groups, HC and PD-MCI groups, PD-NC and PD-MCI groups, respectively, based on the differential results of the digital biomarkers of decision- making in each of these groups.

**FIGURE 3:**
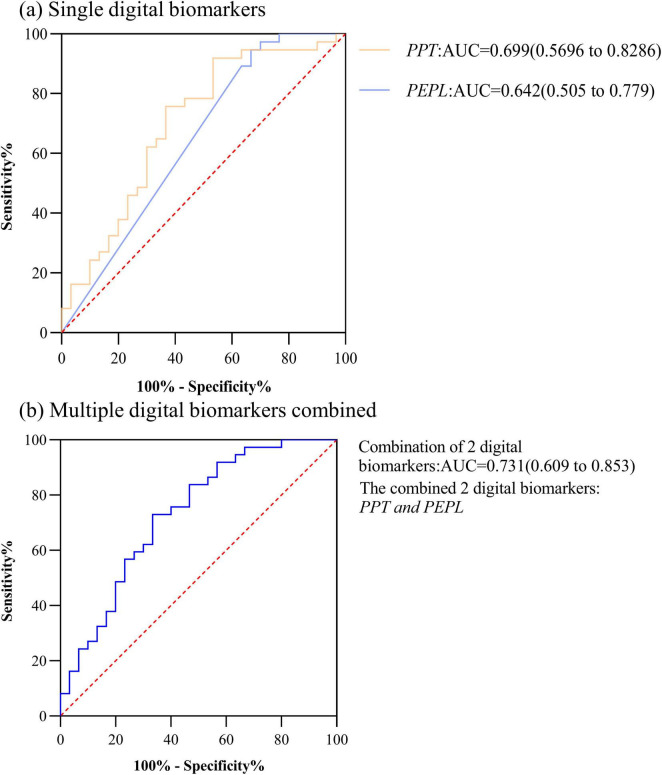
ROC curves, area under the curve, and 95% confidence intervals for differentiating the HC and PD-NC groups. **(a)** Single digital biomarkers; **(b)** Multiple digital biomarkers combined.

In HC and PD-NC groups, the AUC for Performance pause time (*PPT*) was 0.699, the AUC for Performance error path length (*PEPL*) was 0.642, and the AUC for the combination of 2 digital biomarkers was 0.731. The ROC curve, area under the curve, and 95% confidence interval were shown in Figure 3.

**FIGURE 4:**
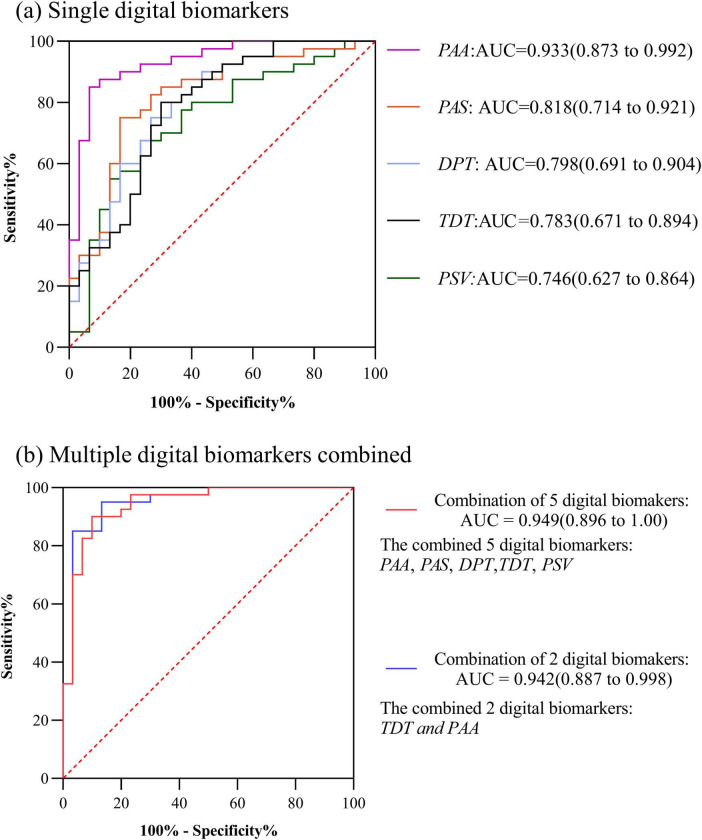
ROC curves, area under the curve, and 95% confidence intervals for differentiating the HC and PD-MCI groups. **(a)** Single digital biomarkers; **(b)** Multiple digital biomarkers combined.

In HC and PD-MCI groups, the AUC for Total decision-making time (*TDT*) was 0.783, the AUC for Decision-making process time (*DPT*) was 0.798, the AUC for Performance average speed (*PAS*) was 0.818,the AUC for Performance speed variability (*PSV*) was 0.746,the AUC for Performance average acceleration (*PAA*) was 0.933. The AUC for the combination of the 5 digital biomarkers was 0.949. However, considering the small sample size included in this study, too many indicator dimensions may be overfitting, so we used stepwise regression to downscale the digital biomarkers, after downsizing, Total decision-making time (*TDT*) and Performance speed variability (*PSV*) were retained, and the AUC for the combination of the 2 digital biomarkers was 0.942. The ROC curve, area under the curve, and 95% confidence interval were shown in Figure 4.

**FIGURE 5:**
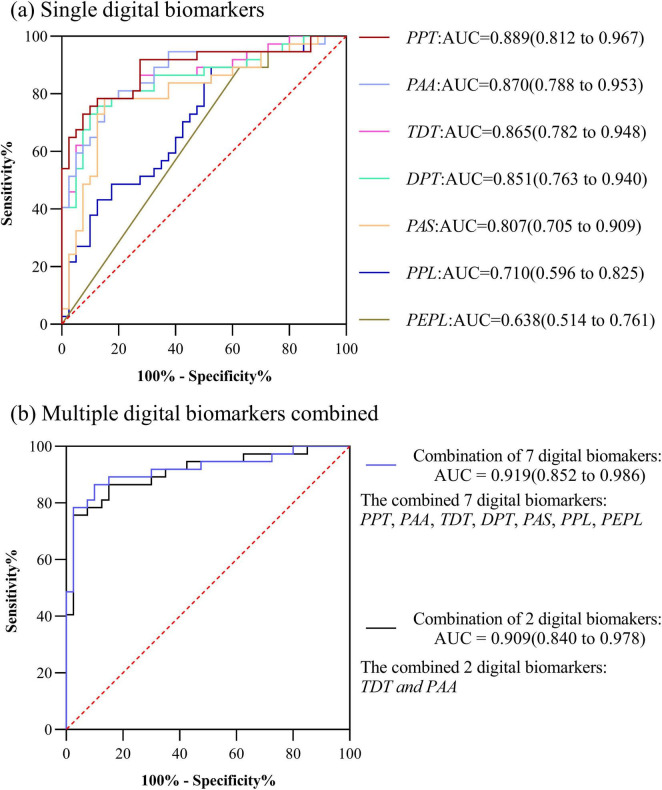
ROC curves, area under the curve, and 95% confidence intervals for differentiating the PD-NC and PD-MCI groups. **(a)** Single digital biomarkers; **(b)** Multiple digital biomarkers combined.

In PD-MCI and PD-NC groups, the AUC for Performance pause time (*PPT*) was 0.889, the AUC for Performance average acceleration (*PAA*) was 0.870, the AUC for Total decision-making time (*TDT*) was 0.865, the AUC for Decision-making process time (*DPT*) was 0.851,the AUC for Performance average speed (*PAS*) was 0.807,the AUC for Performance path length (*PPL*) was 0.710,the AUC for Performance error path length was 0.638. The AUC for the combination of above 7 digital biomarkers was 0.919. However, considering the small sample size included in this study, too many indicator dimensions might be overfitting, so we used stepwise regression to downscale the digital biomarkers, and after downscaling, Performance average acceleration (*PAA*) and Total decision-making time (*TDT*) were retained, and the AUC for the combination of 2 digital biomarkers was 0.909. The ROC curve, area under the curve, and 95% confidence interval were shown in Figure 5.

### 3.4 ROC curves for recognizing PD-MCI in subjects of different genders

We compared the digital decision-making biomarkers in the HC, PD-NC, and PD-MCI groups in different genders. We found that a total of six decision-digital biomarkers were significantly different (*p* < 0.05) between the HC group, the PD-NC group, and the PD-MCI group, and the results of the analysis of specific baseline information were shown in Tables 6, 7, and digital decision-making biomarkers differences were shown in Tables 8, 9.

**TABLE 6:** Distribution of demographic and clinical characteristics of the HC_1_, PD-NC_1_ and PD-MCI_1_ groups.

	HC_1_ *n* = 10	PD-NC_1_ *n* = 21	PD-MCI_1_ *n* = 22	HC_1_ *VS.* PD-NC_1_	HC_1_ *VS.* PD-MCI_1_	PD-NC_1_ *VS.* PD-MCI_1_	HC_1_ *VS.* PD-NC_1_ *VS.* PD-MCI_1_
				***p*-value**			
Age, years	66.20 ± 9.57	63.00 ± 6.28	64.91 ± 8.15	>0.05	>0.05	>0.05	0.522
Years of education	8.50 (4.25)	12.00 (6.00)	11.50 (4.50)	>0.05	>0.05	>0.05	0.222
MMSE score	27.50 (3.00)	28.00 (1.00)	25.00 (2.00)	1.000	0.001**	<0.001**	<0.001**
MoCA score	25.00 (2.00)	25.00 (2.00)	22.00 (2.25)	0.001**	<0.001**	0.084	<0.001**
MDS-UPDRS-III score	4.00 (3.25)	15.00 (4.50)	19.00 (11.50)	1.000	0.001**	<0.001**	<0.001**

**indicates a significant difference between the two groups, at *p* < 0.01.

**TABLE 7:** Distribution of demographic and clinical characteristics of the HC_0_, PD-NC_0_ and PD-MCI_0_ groups.

	HC_0_ *n* = 20	PD-NC_0_ *n* = 16	PD-MCI_0_ *n* = 18	HC_0_ *VS.* PD-NC_0_	HC_0_ *VS.* PD-MCI_0_	PD-NC_0_ *VS.* PD-MCI_0_	HC_0_ *VS.* PD-NC_0_ *VS.* PD-MCI_0_
				***p*-value**			
Age, years	66.50 ± 11.81	64.63 ± 7.94	65.39 ± 6.51	>0.05	>0.05	>0.05	0.839
Years of education	9.25 ± 3.89	10.81 ± 3.89	9.67 ± 4.52	>0.05	>0.05	>0.05	0.517
MMSE score	26.00 (3.75)	28.00 (2.00)	25.00 (2.25)	0.087	0.045*	<0.001**	<0.001**
MoCA score	24.00 (2.75)	25.00 (1.00)	21.50 (4.00)	0.265	0.023*	<0.001**	<0.001**
MDS-UPDRS-III score	4.00 (3.00)	15.00 (6.00)	18.50 (12.50)	<0.001**	<0.001**	1.000	<0.001**

*Indicates a significant difference between the two groups, at 0.01 < *p* < 0.05;

**indicates a significant difference between the two groups, at *p* < 0.01.

**TABLE 8:** Intergroup variability of digital decision-making biomarkers in HC_1_, PD-NC_1_ and PD-MCI_1_ groups.

	HC_1_ *n* = 10	PD-NC_1_ *n* = 21	PD-MCI_1_ *n* = 22	HC_1_ *VS.* PD-NC_1_	HC_1_ *VS.* PD-MCI_1_	PD-NC_1_ *VS.* PD-MCI_1_
				***p*-value**		
**Decision-making global digital biomarkers**						
*TDT, s*	26.40 (19.41)	24.40 (8.65)	46.92 (21.32)	1.000	0.004**	<0.001**
*DPT, s*	27.02 ± (11.40)	23.07 ± (9.63)	42.98 ± (13.81)	1.000	0.003**	<0.001**
**Decision-making planning process digital biomarkers**						
*ILT, s*	0.03 (0.02)	0.02 (1.96)	0.02 (0.02)	>0.05	>0.05	>0.05
*PPT, s*	0.66 (1.81)	0.17 (0.86)	2.44 (2.14)	0.604	0.016*	<0.001**
**Decision-making performance process digital biomarkers**						
*PPL, px*	6581.48 (1680.10)	6660.20 (593.88)	7113.45 (1204.59)	1.000	0.378	0.016*
*PEPL, px*	0.00 (680.50)	0.00 (0.00)	0.00 (352.63)	0.306	1.000	0.056
*NPE, times*	4.00 (7.25)	2.00 (7.00)	1.50 (5.00)	>0.05	>0.05	>0.05
*PAS, px/s*	332.11 (206.49)	309.07 (179.51)	172.08 (88.56)	1.000	0.025*	0.001**
*PSV*	360.07 (175.77)	261.40 (210.31)	209.00 (139.08)	>0.05	>0.05	>0.05
*IPA, px/s2*	0.00 (881.60)	1149.35 (6737.62)	175.41 (1224.55)	0.059	1.000	0.144
*PAA, px/s2*	8565.21 (4328.25)	8432.18 (4167.29)	3953.88 (1375.38)	1.000	<0.001**	<0.001**
*PAA, px/s2*	8565.21 (4328.25)	8432.18 (4167.29)	3953.88 (1375.38)	1.000	<0.001**	<0.001**

*Indicates a significant difference between the two groups, at 0.01 < *p* < 0.05;

**indicates a significant difference between the two groups, at *p* < 0.01.

**TABLE 9:** Intergroup variability of digital decision-making biomarkers in HC_0_, PD-NC_0_ and PD-MCI_0_ groups.

	HC_0_ *n* = 20	PD-NC_0_ *n* = 16	PD-MCI_0_ *n* = 18	HC_0_ *VS.* PD-NC_0_	HC_0_ *VS.* PD-MCI_0_	PD-NC_0_ *VS.* PD-MCI_0_
				***p*-value**		
**Decision-making global digital biomarkers**						
*TDT, s*	29.71 (24.89)	24.81 (17.00)	40.71 (21.15)	1.000	0.045*	0.004**
*DPT, s*	27.82 (21.87)	24.80 (17.00)	40.68 (21.16)	1.000	0.004**	0.006**
**Decision-making planning process digital biomarkers**						
*ILT, s*	0.03 (0.03)	0.02 (0.01)	0.02 (0.02)	>0.05	>0.05	>0.05
*PPT, s*	1.89 (3.91)	0.37 (0.68)	1.93 (2.22)	0.076	1.000	0.012*
**Decision-making performance process digital biomarkers**						
*PPL, px*	7243.75 (2092.82)	6595.54 (647.54)	6731.69 (1075.29)	>0.05	>0.05	>0.05
*PEPL, px*	0.00 (1169.30)	0.00 (0.00)	0.00 (898.34)	>0.05	>0.05	>0.05
*NPE, times*	2.00 (3.75)	1.00 (5.50)	1.00 (5.50)	>0.05	>0.05	>0.05
*PAS, px/s*	258.26 (170.03)	266.44 (152.79)	170.53 (58.14)	1.000	0.001**	0.012*
*PSV*	451.25 (594.12)	330.93 (132.60)	254.65 (140.56)	>0.05	>0.05	>0.05
*IPA, px/s2*	43.93 (2590.04)	462.54 (2450.77)	1267.97 (2643.40)	>0.05	>0.05	>0.05
*PAA, px/s2*	7977.72 (4924.50)	5840.77 (5250.32)	4222.45 (1543.66)	0.630	<0.001**	0.020*

*Indicates a significant difference between the two groups, at 0.01 < *p* < 0.05;

**indicates a significant difference between the two groups, at *p* < 0.01.

We then plotted ROC curves for digital decision-making biomarkers with differentiation for PD-MCI, PD-NC, and HC groups in different genders. Between HC_1_ and PD-MCI_1_ groups in male subjects, the AUC for Total decision-making time (*TDT*) was 0.855, the AUC for Decision-making process time (*DPT*) was 0.818,the AUC for Performance pause time (*PPT*) was 0.832,the AUC for Performance average speed (*PAS*) was0.782,the AUC for Performance average acceleration (*PAA*)was 0.982. Between PD-NC_1_ and PD-MCI_1_ groups in male subjects, the AUC for Total decision-making time (*TDT*) was 0.909,the AUC for Decision-making process time (*DPT*) was 0.890,the AUC for Performance Pause Time (*PPT*) was 0.942,the AUC for Performance path length (*PPL*) was 0.751,the AUC for Performance average speed (*PAS*) was 0.831,the AUC for Performance average acceleration (*PAA*) was0.933. The details were shown in Table 10.

**TABLE 10:** ROC data for HC_1_, PD-NC_1_ and PD-MCI_1_ groups.

	HC_1_ *VS.* PD-MCI_1_		PD-NC_1_ *VS.* PD-MCI_1_	
	**AUC (95% CI)**	***p*-value**	**AUC (95% CI)**	***p*-value**
*TDT, s*	0.855 (0.713–0.996)	0.002	0.909 (0.823–0.995)	<0.001
*DPT, s*	0.818 (0.658–0.978)	0.004	0.890 (0.793–0.986)	<0.001
*PPT, s*	0.832 (0.665–0.999)	0.003	0.942 (0.878–1.000)	<0.001
*PPL, px*	/		0.751 (0.602–0.900)	0.005
*PAS, px/s*	0.782 (0.596–0.967)	0.012	0.831 (0.702–0.960)	<0.001
*PAA, px/s2*	0.982 (0.946–1.000)	<0.001	0.933 (0.845–1.000)	<0.001

Between HC_0_ and PD-MCI_0_ groups in female subjects, the AUC for Total decision-making time (*TDT*) was 0.736,the AUC for Decision-making process time (*DPT*) was 0.797,the AUC for Performance average speed (*PAS*) was 0.850,the AUC for Performance average acceleration (*PAA*) was 0.889. Between PD-NC_0_ and PD-MCI_0_ groups in female subjects, the AUC for Total decision time (*TDT*) was 0.816,the AUC for Decision-making process time (*DPT*) was 0.816,the AUC for Performance pause time (*PPT*) was 0.819,the AUC for Performance average speed (*PAS*) was 0.781,the AUC for Performance average acceleration (*PAA*) was 0.778. The details were shown in Table 11.

**TABLE 11:** ROC data for HC_0,_ PD-NC_0_ and PD-MCI_0_ groups.

	HC_0_ *VS.* PD-MCI_0_		PD-NC_0_ *VS.* PD-MCI_0_	
	**AUC (95% CI)**	***p*-value**	**AUC (95% CI)**	***p*-value**
*TDT, s*	0.736 (0.576–0.896)	0.013	0.816 (0.663–0.969)	0.002
*DPT, s*	0.797 (0.656–0.939)	0.002	0.816 (0.663–0.969)	0.002
*PPT, s*	/		0.819 (0.658–0.981)	0.002
*PAS, px/s*	0.850 (0.727–0.973)	<0.001	0.781 (0.619–0.944)	0.005
*PAA, px/s2*	0.889 (0.784–0.994)	<0.001	0.778 (0.620–0.935)	0.006

## 4 Discussion

In this study, we proposed a novel method to evaluate the cognitive deficits in decision-making of PD-MCI, which is suitable for application in community scenarios, and is characterized by short time-consumption, high sensitivity, and low cost. The method is based on an independently designed maze decision-making digital evaluation paradigm, which characterizes and quantifies the cognitive deficits of decision in PD-MCI population at a fine-grained level. We extracted digital decision-making biomarkers that were differentiated between the PD-MCI, PD-NC and HC groups, respectively. Due to the limited sample size and in order to avoid overfitting, we used stepwise regression to downscale the above digital biomarkers of decision making with variability between groups. The final 2 digital decision-making biomarkers Performance average acceleration (*PAA*) and Total decision-making time (*TDT*), were retained in the PD-MCI group versus the PD-NC group, and in the PD-MCI group versus the NC group, and their combined screening efficacy AUC was 0.909 and 0.942 respectively.

The diversity of cognitive impairment patterns in PD-MCI patients requires a comprehensive assessment with multiple single-domain screening scales, but this process is highly subjective, time-consuming, and of low accuracy. It has been found that decision-making, as a window to cognition (Shadlen and Kiani, 2013), is the result of a combination of multiple cognitive abilities with computable and interpretable properties. The maze decision-making paradigm designed in this study, in which subjects need to make a path prediction through visual search or selective attention, involves multiple cognitive functions such as visuo-spatial, executive, and attention. Therefore, it is important to characterize the cognitive decline of PD-MCI based on decision-making tasks for identification and monitoring.

First, the results of this study confirmed that there was no statistical difference in the MDS-UPDRS-III part scores between the PD-MCI group and the PD-NC group; however, the Performance average acceleration (*PAA*) and the Performance average speed (*PAS*) of the PD-MCI group were smaller than those of the PD-NC group, and the total decision-making time (*TDT*), the Decision-making process time (*DPT)*, and the Performance pause time (*PPT*) were larger than that of the PD-NC group; similarly, it was confirmed that the Performance average acceleration (*PAA*) and the Performance average speed (*PAS*) of the PD-MCI group were smaller than that of the HC group, and that the Total decision-making time (*TDT*) and Decision-making process time (*DPT*) of the PD-MCI group were larger than that of the HC group, and the above results were basically in line with the conclusions of the previous studies (Galtier et al., 2019; Schalkamp et al., 2023). However, compared to the previous studies, in the present study, the speed, time, and other metrics were extracted in the cognitive task state, not purely the movement data in daily life. Executive speed, acceleration, and time are important in responding to the cognitive function of decision-making. They reflect the individual’s information processing efficiency and strategy selection in the decision-making process and reveal the temporal dynamics of the decision-making process. By considering these metrics together, a deeper understanding of the behavioral performance and decision-making process of PD-MCI individuals in the face of complex decision-making tasks can be achieved. Therefore, the digital decision-making biomarkers collected in this study can well respond to the cognitive functions of the PD-MCI population.

Previous studies have also shown that patients with PD-MCI have deficits in executive functioning and decision-making cognitive functioning compared with normal controls or PD-NC (Martin et al., 2013; Pan et al., 2022). Decision-making, as a window to cognition, is the result of a combination of cognitive abilities. In the field of brain science research, the frontal cortex is recognized as the main brain structure associated with decision-making abilities, especially the dorsolateral prefrontal cortex, and the ventral lateral prefrontal cortex, which are involved in cognitive processes such as cognitive flexibility, working memory, acquisition, and stimulus-reward (Friedman and Robbins, 2022; Zhang et al., 2022). In recent years it has been recognized that decision-making is not only dependent on the frontal cortex, but is also closely related to several other executive neural network brain regions that are closely linked to the frontal cortex. For example, the frontal cortex is closely connected to brain regions such as the striatum (Brockett et al., 2022), hippocampus (Shintaki et al., 2024), and hypothalamus (Chen et al., 2024), and these connections are critical for the regulation of auditory memory and goal-directed behavior. In addition, navigational ability refers to an individual’s decision-making ability to orient, localize, and plan a path through an environment. The neural basis for flexible navigation has long been focused on hippocampal formation, but recent evidence suggests that subregions of prefrontal cortex are critical for many aspects of navigation, especially in complex or dynamic environments (Patai and Spiers, 2021). Among these, the dorsolateral prefrontal cortex and the ventral lateral prefrontal cortex act as inhibitors and replanners in tortuous paths such as mazes; the dorsolateral anterior cingulate cortex is associated with planning and spontaneous internal route changes; and the orbitofrontal cortex integrates environmental representations with action values to provide a holistic decision map of possible decisions (Shadlen and Kiani, 2013). It has been demonstrated that prefrontal damage exists in PD-MCI patients (Hirano, 2021; Zarifkar et al., 2021). Therefore, the PD-MCI group may have deficits in decision-making cognitive functioning due to damage to the prefrontal cortex, and consequently abnormal digital decision-making biomarkers, during the maze decision-making digital assessment paradigm.

Notably, we found that most of the digital decision-making biomarkers, such as Performance average speed (*PAS*), Performance average acceleration (*PAA*), and Total decision-making time (*TDT*), in the population of the PD-NC group did not differ from that of the HC group. These results are inconsistent with previous studies that concluded that the speed or acceleration of PD was greater than that of normal controls (Galtier et al., 2019; Schalkamp et al., 2023). The reason for this may be that the previous research paradigm was mostly a single motor task that did not require complex decision-making and thinking, whereas the assessment paradigm in our study was a complex decision-making task with a lot of constraints on rules that were more cognitive in nature. The paradigm in our study was a complex decision-making task with more rule constraints and more cognitive functions were examined. Therefore, the paradigm in this study was designed to characterize the cognitive functions of decision-making in PD-MCI.

In addition, considering gender as an important variable in PD-MCI, this study explored the variability of decision digital biomarker screening to distinguish PD-MCI, PD-NC, and HC among different genders. Among the different genders, there were no statistically differentiated decision digital biomarkers in PD-NC and HC, but there were statistically differentiated decision digital biomarkers in PD-MCI and PD-NC, and PD-MCI and HC. Previous studies have suggested that a possible source of variability in cognitive traits in PD is the effect of estrogen on dopaminergic neurons and pathways in the brain (Miller and Cronin-Golomb, 2010). The results of the present study showed that the decision process time (DPT), the Performance average speed (*PAS*), and the ability to discriminate between PD-MCI and PD-NC were comparable across genders but that the Total decision-making time (*TDT*), the Performance average acceleration (*PAA*) and the Performance pause time (*PPT*) were significantly higher in males than in females, suggesting that deficits in decision-making executive function may be more pronounced in males relative to females with PD-MCI; analyzing the reasons for this, it may be related to the fact that estrogen has a neuroprotective effect on the brain’s dopaminergic pathway (Liu and Dluzen, 2007). Also across genders, the Decision-making process time (*DPT*), the Performance average speed (*PAS*), and the ability to discriminate between PD-MCI and HC were comparable, but the Total decision-making time (*TDT*), Performance average acceleration (*PAA*) were significantly higher in men than in women. However, given the small sample size after grouping, the above results need a larger sample size to give validation.

Second, many previous studies have also used the maze digital assessment paradigm for screening and identification of PD. However, there is a gap in studies related to the digital maze task and the quantification of the whole process of the maze task process at a fine-grained level and its use to explore cognitive dysfunction in patients with PD-MCI. Some of the studies on the application of digital maze tasks to PD in recent years are shown in Table 12. Compared with previous studies, the maze paradigm of the present study differed in the following ways: (1) In terms of participant recruitment: compared to the PD patients included in the above studies, the PD patients included in this study were those who were clear about whether or not they were accompanied by cognitive impairment, with relatively little heterogeneity, and differentiated between PD-NC and PD-MCI, and between PD-MCI and HC patients, with a specificity and sensitivity of up to 0.9, which provided a high early warning efficacy; (2) In terms of maze design: This study refers to the maze generation website (see text footnote 1) created by John Lauro and other researchers at the University of Michigan as the maze layout generator, and also refers to the relevant studies on the assessment of cognitive status of middle-aged and elderly people with neurodegenerative disorders using the maze test (Nef et al., 2020). Based on previous studies and daily life observations (Li et al., 2022), the majority of subjects were right-handed, so a maze from the lower-right entrance to the upper-left exit was selected so as not to interfere with the subject’s vision during operation. As a common tool for testing cognitive ability, spatial memory, and learning ability, mazes have corresponding design criteria (McClendon, 2001). According to the design principles of the perfect maze (Bellot et al., 2021), ① the correct path accounts for more than half of the total path length of the maze; ② there is a forward dead end (i.e., the same direction as the correct direction); ③ turns can be seen in 40% of the correct paths; ④ there are decision points (T-junctions and intersections) in 2% of the correct paths; and ⑤ there are average decision points (T-type intersections and crossroads) in 2% of the correct paths. and intersections); ⑥ on average, each forward dead end has 40% of turns (corners) and 1% of decision points (T-intersections and intersections). Therefore, the difficulty of the maze paradigm designed in this study is more reasonable and standardized. (3) In terms of digital biomarkers (indicators) extraction: this study quantifies the decision-making behaviors of participants in the process of paradigm evaluation at a fine-grained level, which is more comprehensive compared to the indicator dimensions extracted in the above studies. In this study, two dimensions of decision-making global digital biomarkers and decision-making process digital biomarkers were extracted, in which the decision-making process digital biomarkers were divided into decision-making planning process digital biomarkers and decision execution process digital biomarkers. In summary, the innovation of this study is based on cognitive computational neuroscience, designing a digital maze paradigm with a high degree of standardization, quantifying the decision-making behaviors of PD-MCI patients throughout the entire process of the task, and extracting digital decision-making biomarkers that can characterize cognitive deficits in PD-MCI patients, which will provide a new way of thinking about large-scale screening in the community.

**TABLE 12:** Selected studies of digital maze tasks applied to PD in recent years.

Researcher	Method	Limitation
Schneider et al., 2017	In this study, a digitized virtual water maze test was designed to extract metrics including heading error, path length, and latency to locate the target.	(1) The small PD-MCI sample size of 12; (2) the spatial learning of all included PD patients was severely impaired, and the paradigm failed to differentiate between patients with normal PD cognition and those with impaired PD-MCI cognition; and (3) the limited number of dimensions for which the metrics were extracted.
Virmani et al., 2023	This maze game simulates gait by moving a frog through a maze environment using bipedal gait-like bimanual movements, extracting gait stride length, stride time, stride width, and stride speed to quantify how well subjects performed on the task.	(1) 27 PD patients were included and most of them had motor impairments; (2) the maze complexity was low and the standardization was limited; and (3) The extracted metrics were mainly based on the gait characteristics of PD and the metrics were of limited dimensions.
Nef et al., 2020	The NL maze game, which requires participants to complete the maze in the shortest possible time with the fewest number of steps, is used to assess changes in visuomotor, visual structural, and executive functions in neurodegenerative diseases.	(1) Primarily assessed cognitive and motor function in aging and neurodegenerative diseases and with only four PDs; and (2) the paradigm was designed with a single difficulty level design and limited response to the individual’s level of cognitive ability.
Zeng et al., 2003	The Kiel Motion Maze automatically records different types of spatial memory errors, distances and rotation angles, decision times and reaction times for each move.	(1) The sample size of PD non-demented patients was only 16 and did not specifically identify their cognitive functional status; (2) The design was more complex and time-consuming, taking half an hour to complete in some patients.
Leplow et al., 2002	A search motor task combining basic features of the radial arm maze and morris water maze paradigms was used. Participants had to find and memorize 5 of 20 hidden locations in a fully controlled environment to explore spatial behavior in mildly impaired PD.	(1) The small sample size of 14 PDs included did not specifically identify their cognitive functional status; and (2) The procedure was relatively complex and poorly adapted for aging.

Finally, there are some limitations to this study. (1) Only 107 subjects were included in this study, which is a small sample size, all from one medical center, and the results of the study may not reflect the general population of PD-MCI. In the future, we will increase the sample size by collecting data through the cooperation of multiple hospitals to improve the accuracy of the study results. (2) Although digital biomarkers characterized and quantified by the maze decision-making digital evaluation paradigm show good screening efficacy, more research is needed to gain insights into the physiological, pathological, or anatomical characterization of these digital biomarkers, and in particular, to explore the functional brain connectivity or brain network mechanisms behind these digital biomarkers based on fMRI. (3) Due to the limitations of the study conditions, we only conducted a cross-sectional study and included only PD-MCI, PD-NC, and HC; in the future, we will try to include some diseases that are likely to be confused with PD-MCI, such as patients with MCI due to AD, and conduct a longitudinal study to further improve the validity and reliability of this paradigm.

## 5 Conclusion

In summary, we explored the cognitive deficits in decision-making in the PD-MCI population by using an independently designed maze decision-making digital assessment paradigm, and mined novel digital biomarkers for identifying early cognitive decline in PD-MCI. The method was clinically validated with a screening efficacy AUC of 0.909 for PD-MCI and PD-NC, and 0.942 for PD-MCI and NC, as well as the advantages of rapidity, inexpensiveness, and high level of objectivization, which provide new ideas for the study of the mechanism of PD decision-making deficits and the rapid digital assessment of cognitive impairment in PD.

## Data Availability

The raw data supporting the conclusions of this article will be made available by the authors, without undue reservation.

## Footnotes

1
http://hereandabove.com/maze/mazeorig.form.html
