# Supplementary material for: A study on the exploration of mild cognitive impairment in Parkinson’s disease based on decision-making cognitive computing
Source: Front Neurosci. 2025 Jan 7;18:1495975. doi: 10.3389/fnins.2024.1495975 (PMC11747548; doi:10.3389/fnins.2024.1495975)
Supplement: Supplementary file 1 [file Data_Sheet_1.docx]

Supplementary Material

# Supplementary Data

(1). Demographic and Clinical Characteristics

Before the formal experiment started, we conducted a pre-trial. In the pre-experiment, a total of 50 subjects aged 45-85 years, including 20 patients with PD-MCI, 15 patients with PD-NC, and 15 normal elderly people, were enrolled in the PD-MCI group, the PD-NC group, and the HC group, respectively. We analyzed the baseline information of the three groups for differences in terms of age, gender, years of education, MMSE scales, MoCA scales, and MDS-UPDRS scales of the subjects in the three groups, and the results of the analysis of differences in the three groups are shown in Table 1.

(2) Analysis of Digital Biomarkers

We compared the decision-making digital biomarkers in the HC group, the PD-NC group, and the PD-MCI group. We found that a total of five decision-making digital biomarkers were significantly different between the HC group, the PD-NC group, and the PD-MCI group (p < 0.05), and the results of the specific decision-making digital biomarker difference analysis are shown in Table 2.

(3) ROC Curves for Identifying PD-MCI Patients from All Participants

We performed subject work characteristics (ROC) curve analyses for HC VS. PD-MCI, PD-NC VS. PD-MCI, respectively, based on the differential results of decision-making digital biomarkers in each of these groups.

Where the AUC of Performance average acceleration (PAA) is 0.923, the AUC of Decision process time (DPT) is 0.813, the AUC of Total decision time (*TDT*) is 0.803, and the AUC of Performance average speed (*PAS*) is 0.793. where the AUC of Performance pause time (*PPT*) has an AUC of 0.880, Performance average acceleration (*PAA*) has an AUC of 0.877, Total decision time (*TDT*) has an AUC of 0.830, and Decision process time (*DPT*) has an AUC of 0.800. as shown in Table 3.

# 2.Supplementary table

|  | HC n = 15 | PD-NC n = 15 | PD-MCI n = 20 | HC *VS.*  PD-NC | HC *VS.*  PD-MCI | PD-NC *VS.* PD-MCI | HC *VS.* PD-NC *VS.* PD-MCI |
| --- | --- | --- | --- | --- | --- | --- | --- |
|  |  |  |  | *p* value | | | |
| Age, years | 66.13 ± 11.61 | 62.40 ± 7.26 | 66.10 ± 6.74 | >0.05 | >0.05 | >0.05 | 0.300 |
| Sex (female/ male) | 7/8 | 7/8 | 8/12 | >0.05 | >0.05 | >0.05 | 0.897 |
| Years of education | 9.00(5.00) | 12.00(6.00) | 11.50(6.75) | >0.05 | >0.05 | >0.05 | 0.245 |
| MMSE score | 27.00(3.00) | 28.00(2.00) | 25.00(1.75) | 0.395 | 0.003** | ＜0.001** | ＜0.001** |
| MoCA score | 24.27 ± 2.34 | 25.13 ± 0.99 | 21.55 ± 2.33 | 0.744 | 0.001** | ＜0.001** | ＜0.001** |
| MDS-UPDRS-Ⅲ score | 4.00(3.00) | 14.00(4.00) | 17.00(8.75) | 0.001** | 0.001** | 0.480 | ＜0.001** |

**Table 1** Distribution of demographic and clinical characteristics of the HC, PD-NC and PD-MCI groups.

Note: * indicates a significant difference between the two groups, at *p*<0.05;

** indicates a significant difference between the two groups, at *p*<0.01.

**Table 2** Differential results of decision-making digital biomarkers in HC, PD-NC and PD-MCI groups.

|  | HC n = 15 | PD-NC n = 15 | PD-MCI n = 20 | HC VS. PD-NC | HC VS. PD-MCI | PD-NC VS. PD-MCI |
| --- | --- | --- | --- | --- | --- | --- |
|  |  |  |  | p value | | |
| Decision-making global digital biomarkers | | | | | | |
| DT, s | 27.31(22.37) | 24.40(17.10) | 43.48(19.28) | 1.000 | 0.007** | 0.003** |
| DPT, s | 26.82 ± 11.90 | 27.44 ± 12.99 | 44.52 ± 17.21 | 1.000 | 0.003** | 0.004** |
| Decision-making planning process digital biomarkers | | | | | | |
| ILT, s | 0.03(0.02) | 0.02(0.02) | 0.02(0.02) | ＞0.05 | ＞0.05 | ＞0.05 |
| PPT, s | 0.94(2.02) | 0.24(0.65) | 1.93(3.32) | 0.148 | 0.218 | ＜0.001** |
| Decision-making performance process digital biomarkers | | | | | | |
| PPL, px | 6607.79(1780.83) | 6745.05(732.68) | 6921.02(2701.11) | ＞0.05 | ＞0.05 | ＞0.05 |
| PEPL, px | 0.00(1002.45) | 0.00(0.00) | 0.00(1310.07) | ＞0.05 | ＞0.05 | ＞0.05 |
| NPE, times | 2.00(6.00) | 1.00(4.00) | 3.00(6.25) | ＞0.05 | ＞0.05 | ＞0.05 |
| PAS, px/s | 330.90(202.15) | 290.55(206.33) | 178.01(86.33) | 1.000 | 0.011* | 0.063 |
| PSV | 361.50(238.14) | 342.80(256.39) | 242.33(146.05) | ＞0.05 | ＞0.05 | ＞0.05 |
| IPA, px/s^2^ | 700.12(2609.97) | 1988.03(7848.22) | 802.45(2316.71) | ＞0.05 | ＞0.05 | ＞0.05 |
| PAA, px/s^2^ | 8616.78(4250.30) | 8329.14(7168.43) | 4081.58(1625.36) | 1.000 | ＜0.001** | ＜0.001** |

Note: * indicates a significant difference between the two groups, at *p*<0.05;

** indicates a significant difference between the two groups, at *p*<0.01

**Table 3** ROC data for HC, PD-NC and PD-MCI groups.

|  | HC VS. PD-MCI | | PD-NC VS. PD-MCI | |
| --- | --- | --- | --- | --- |
|  | AUC (95% CI) | p value | AUC (95% CI) | p value |
| TDT, s | 0.803 (0.652-0.955) | 0.002 | 0.830 (0.687-0.973) | 0.001 |
| DPT, s | 0.813 (0.671-0.956) | 0.002 | 0.800 (0.646-0.954) | 0.003 |
| PPT, s | / | | 0.880 (0.751-1.000) | <0.001 |
| PAS, px/s | 0.793 (0.637-0.949) | 0.003 | / | |
| PAA, px/s^2^ | 0.923 (0.828-1.000) | <0.001 | 0.877 (0.747-1.000) | <0.001 |
